# Quality of life of People living with HIV and AIDS attending the Antiretroviral Clinic, University College Hospital, Nigeria

**DOI:** 10.4102/phcfm.v4i1.294

**Published:** 2012-02-16

**Authors:** Oluyemisi F. Folasire, Achiaka E. Irabor, Ayorinde M. Folasire

**Affiliations:** 1 Department of Family Medicine, University of Ibadan, Nigeria

## Abstract

**Background:**

Quality of life (QOL) is an important component in the evaluation of the well-being of people living with HIV and AIDS (PLWHA), especially with the appreciable rise in longevity of PLWHA. Moreover, limited studies have been conducted in Nigeria on how PLWHA perceive their life with the World Health Organisation Quality of Life Brief Scale (WHOQOL-Bref) instrument.

**Objective:**

This study assessed the QOL of PLWHA attending the antiretroviral (ARV) clinics, UCH Ibadan, Nigeria.

**Method:**

A cross-sectional study was conducted from June to September 2008 that involved 150 randomly selected HIV-positive patients who were regular attendees at the antiretroviral clinic, UCH Ibadan. An interviewer administered questionnaire was used to collect information on sociodemographic data, satisfaction with perceived social support, medical records, and QOL was assessed with WHOQOL-Bref.

**Results:**

The mean age of the respondents was 38.1 ± 9.0 years and the male: female ratio was 1:2. The mean CD4 count was higher in female patients than in male patients, 407 cells/mm^3^: 329 cells/mm^3^ (*p* = 0.005). The mean QOL scores on the scale of (0–100) in three domains were similar: psychological health, 71.60 ± 18.40; physical health, 71.60 ± 13.90; and the environmental domain, 70.10 ± 12.00; with the lowest score in the social domain, 68.89 ± 16.70. Asymptomatic HIV-positive patients had significantly better mean QOL scores than symptomatic patients in the physical (74.04 ± 16.85 versus 64.47 ± 20.94, *p* = 0.005) and psychological domains (76.09 ± 12.93 versus 69.74 ± 15.79, *p* = 0.015). There was no significant difference in the mean QOL scores of men compared to those of women, in all domains assessed.

**Conclusion:**

High QOL scores in the physical, psychological and environmental domains may be reflective of the effectiveness of some of the interventions PLWHA are exposed to at the ARV clinic, UCH Ibadan (on-going psychotherapy, free antiretroviral drugs). Relatively low social domain scores may suggest ineffective social support networks, because PLWHA are still exposed to stigmatisation and discrimination. An improvement in social support for PLWHA, therefore, will improve their quality of life further.

## Abstrait

**Qualité de vie des personnes vivant avec le VIH et le Sida se rendant dans le centre de traitement antirétroviral de l'hôpital universitaire au Nigeria**

**Contexte:**

La qualité de vie (QV) est une composante importante de l’évaluation du bien-être des personnes vivant avec le VIH et le Sida (PVVS), en particulier en raison de l'augmentation sensible de la durée de vie des PVVS. De plus, des études limitées ont été réalisées au Nigeria sur la façon dont les PVVS percevaient leur vie, en utilisant l'instrument de l’Échelle de qualité de vie générique (WHOQOL-Bref) de l'Organisation mondiale de la Santé.

**Objectif:**

Cette étude a évalué la QV des PVVS se rendant dans le centre de traitement antirétroviral (ARV), CHU d'Ibadan, Nigeria.

**Méthode:**

Une étude transversale a été réalisée de juin à septembre 2008 portant sur 150 patients séropositifs sélectionnés de manière aléatoire, qui se rendaient régulièrement au centre de traitement antirétroviral du CHU Ibadan. Un questionnaire administré par des enquêteurs a été utilisé afin de recueillir des informations sur les données sociodémographiques, la satisfaction quant au soutien social perçu, les dossiers médicaux, et la QV a été évaluée avec le WHOQOL-Bref.

**Résultats:**

L’âge moyen des sondés étaient de 38.1 ± 9.0 ans et le ratio hommes/femmes était de 1/2. Le compte de CD4 moyen était plus élevé chez les femmes que chez les hommes, 407 cellules/mm3: 329 cellules/mm3 (*p* = 0.005). Les notes de QV moyennes obtenues sur l’échelle de (0 à 100) dans trois domaines étaient similaires: santé psychologique, 71.60 ± 18.40; santé physique, 71.60 ± 13.90, et le domaine environnemental, 70.10 ± 12.00; la note la plus basse ayant été obtenue dans le domaine social, 68.89 ± 16.70. Les patients séropositifs asymptomatiques avaient des notes de QV plus élevées que les patients symptomatiques dans les domaines physique (74.04 ± 16.85 contre 64.47 ± 20.94, *p* = 0.005) et psychologique (76.09 ± 12.93 contre 69.74 ± 15.79, *p* = 0.015). Aucune différence n'a été observée entre les notes de QV moyennes des hommes et celles des femmes, dans tous les domaines évalués.

**Conclusion:**

Des notes de QV élevées dans les domaines physique, psychologique et environnemental peuvent traduire l'efficacité de certaines interventions dont les PVVS se rendant dans le centre de traitement ARV du CHU d'Ibadan bénéficient (psychothérapie continue, médicaments antirétroviraux gratuits). Les notes relativement basses dans le domaine social peuvent refléter l'inefficacité des réseaux de soutien social, les PVVS faisant toujours l'objet de stigmatisation et de discrimination. Un meilleur soutien social en faveur des PVVS améliorera donc encore davantage leur qualité de vie.

## Introduction

Since the beginning of the pandemic, almost 60 million people have been infected with HIV and 25 million people have died of HIV-related causes. The current HIV and AIDS epidemic projections indicate that, as of the end of 2009, 33.3 million people worldwide were living with HIV; 2.6 million more people were infected in 2009, and 1.8 million people had lost their lives to AIDS in the same year.^1, 2^

An estimated 22.5 million people were living with HIV and AIDS in sub-Saharan Africa as of the end of 2009. Nigeria ranks second in sub-Saharan Africa because of her large population of approximately 152.9 million.^3^ Since the first report of HIV and AIDS in Nigeria in 1986, the national prevalence from antenatal surveys demonstrated a steady increase from 1.8% in 1991 to 4.5% in 1995, and peaked at 5.8% in 2001; after that it started to decline slowly to 4.4% in 2005. The national prevalence appeared to be stabilising around 4.4% – 4.1% from 2005 to 2010.^3, 4^ These findings could be a result of the positive effect of various interventions to prevent the spread of HIV infection, especially amongst the youth, as well as the increased access to ART from the large scale-up programmes across the country, resulting in an improved survival rate amongst the PLWHA. With the current prevalence of 4.1%, it is estimated that more than 3.1 million Nigerians are infected as of the end of 2010.^4^ HIV and AIDS has debilitating effects on all aspects of the lives of individuals and families; ranging from physical health, as well as the psychological, economic and social aspects of life.^3^

Social support refers to the perceived comfort, care, esteem, or help a person receives from other people or groups such as the spouse, lover, friends, family, co-workers, or physicians.^5^ Family members, in particular a spouse, appear to be the most important sources of social support, and account for most of the association between social support and health. There is evidence that support from sources outside the family cannot compensate for what is missing in the family.^6^

In Africa, social services have been underserved because the countries do not give priority to basic infrastructure for their people. Individuals and families affected by a health crisis have no structure to fall back on; therefore, quality of life is seriously affected until the ill family member can be reintegrated fully to societal responsibilities. Chronic illness has always constituted a barrier to quality of life, but in particular with afflictions such as HIV and AIDS. These afflictions are sources of much discrimination and lead to degeneration of family involvement as well as rejection in all probability by the only structure of support available in Africa.

The World Health Organisation (WHO) has defined Quality of Life (QOL) as individuals’ perceptions of their position in life in the context of the culture and value systems in which they live and in relation to their goals, standards, expectations and concerns.^7^, ^8^ This definition emphasises the importance of an overall subjective feeling of well-being pertaining to aspects of morale, happiness and satisfaction. QOL, therefore, relates to both an adequacy of material circumstances as to how satisfied an individual is with these circumstances of life.^9^ It usually refers to the degree to which a person's life is desirable or undesirable.^10^ QOL comprises a collection of interacting objective and subjective dimensions, which may change over time in response to life, health events and experiences. Quality of life can also be explained by asking questions such as, (1) how much is your income? (this suggests objective aspects of QOL) and (2) how satisfied are you with your income? (the question conveys the subjective aspects of QOL). The assessment of QOL as a measure of treatment outcomes has become popular in Medicine because the concept of QOL itself captures exactly the notion that the ultimate goal of medical intervention is to improve the well-being of the patient.^11^

Through reflection on the sense of well-being and satisfaction experienced by people under their current life circumstances, the assessment of QOL aims to provide a comprehensive evaluation of the individual's well-being, which includes an assessment of their role functioning, community integration and personal adjustment.^11^ This implies that HIV and AIDS do not only affect the physical well-being of PLWHA, but also the overall quality of life and the perceptions of various aspects of their lives and daily living.

Several instruments for measuring QOL have been developed and described, but very few studies had used the WHOQOL-Bref (World Health Organisation Quality of Life Brief Scale) instrument in people living with HIV and AIDS.^8, 10, 12–15^ Its documented use in HIV-positive patients in Nigeria is limited and the use of the disease-specific version was reported only recently.^16^

The majority of quality of life studies on PLWHA in developing countries did not use the WHOQOL-Bref; the general opinion from such studies is that sociodemographic and socio-economic variables, as well as the presence, number and severity of HIV symptoms, and the use of highly active antiretroviral therapy (HAART), was reported to have a significant impact on the quality of life of PLWHA.^17–19^ In such studies, it was reported that QOL summarised the judgements people make to describe their experiences of health and illness. These judgements include that expectations are learnt from experiences and are therefore highly specific, and they vary between individuals subject to differences in psychological, economic, demographic, social, and other cultural factors.^19, 20^

The perception of QOL is not a function of only physical health but is dependent on factors such as age, sex, educational level, and income and employment status, independent of the health status. Louwagie and colleagues^18^ reported in a study on HIV-positive patients that unemployed people had lower health index scores than employed people. This could be attributed to the fact that people who are more sick are more likely to quit their job, and not that unemployment itself affected well-being directly. Adedimeji and Odutolu used a qualitative approach to determine the extent to which social, economic, psychological, health-resource and environmental factors contribute to the improvement of the quality of life of PLWHA.^19^ It was assumed that individuals who have access to care, social support and ARV therapy will experience better QOL than those who do not have the access. The study approach generated a pool of ideas of what PLWHA considered important in quality of life. Results of ranking of factors determining their QOL showed that availability of care and social support from family members and close friends are rated the highest (93%); financial pressure (89%), stigma and discrimination (87%), health concerns and counselling (85%) where also rated highly. In the same study^19^ the desire for care and support considerably influenced the willingness to disclose information about the person's HIV status. Where support is not expected, information about the HIV-positive status is withheld.

The provision of care and support is largely influenced by the prevailing cultural norms amongst the people concerned who are burdened with the task of providing care for those who are ill. Given the ignorance and stigma that surround HIV and AIDS, such care is provided only by close relatives of PLWHA who consider it an obligation to take care of their own; however, it is important to note that such level of care is not universal for all PLWHA. Some respondents reported that they were isolated and rejected by the community and family members. The findings of Adedimeji et al.^19^ confirm earlier studies^21^ that psychosocial factors and social support do influence health outcomes of HIV infected individuals. There is evidence that social support affects health outcomes either through its effects on the function of the immune system or through its effects on self-care activities and other illness behaviours. Obtaining social support may, however, be problematic for some persons because of AIDS-related stigma. The findings about the psychosocial effects of testing positive for HIV are important when it is considered that psychological and existential issues impact heavily on quality of life of PLWHA. Constant worry, stress and anxiety contribute to poor QOL, but these could also become catalysts for adopting health enhancing behaviour. Similarly, continued stigma and social isolation still pose considerable barriers to disclosure of HIV status. It has implications for access to treatment facilities, compliance with medication, and generally hinders preventative efforts directed at HIV and AIDS.

Louwagie, in a cross-sectional analytical study on South African adults with HIV infection, compared the health-related quality of life of HIV-positive patients receiving HAART with those who were awaiting treatment, and highlighted the influence of HAART on improving symptoms of HIV infection and quality of life of the respondents.^14^ The study revealed that patients who were receiving HAART reported better QOL scores for most of the domains assessed, and reported significantly less problem scores than those not yet on HAART. It has also been documented that the presence, number, and severity of symptoms are major determinants of quality of life in HIV infected patients, and that worse symptoms are associated with worse perceptions of QOL.^17, 18, 21^

### Justification for the study

This is the first time that quality of life of PLWHA at the ARV clinic, UCH, has been studied. Few of the QOL studies conducted in Ibadan examined the influence of psychiatric morbidities, especially the influence of depression, on QOL. As a result, this study provides baseline information on the perceived QOL of people living with HIV and AIDS with regard to the influence of service delivery and health status as from the time that care and treatment in UCH, Ibadan, were accessed. The perceived quality of life has amongst other effects, social implications on everyone, because a person's perception of well-being will determine the acquisition of habits related to tasks, employment and care of others. HIV and AIDS has such serious repercussions on psychic, social and physical well-being, that the assessment of QOL of people living with HIV and AIDS helps to gauge how these people are re-integrated in society after the initial health crisis they face on diagnosis of this disease, thus enabling them to meet their daily responsibilities. Information such as this is important in the evaluation of the impact of the disease on health outcomes, and the effect of the intervention already on the ground, as well as to plan other interventions that will help to meet other identified needs of PLWHA and improve the quality of life of Nigerians living with HIV and AIDS.

The study was designed to assess how PLWHA who accessed care from the antiretroviral therapy (ARV) clinic of the University College Hospital (UCH) in Ibadan, south-west Nigeria, perceived their quality of life. The ARV clinic located within the premises of UCH attends to patients from Ibadan city which is the largest city within the western states of Nigeria; the clinic receives referrals also from surrounding towns, cities and states, as far away as the eastern and northern parts of Nigeria. This clinic is one of Federal Government designated antiretroviral centres in Nigeria, but PLWHA from far away states still prefer to travel the distance, mainly to protect their privacy; they do not want to be seen accessing HIV care and services in their own domicile.

### Significance of the study

Assessment of quality of life has become an important outcome measure in the management of chronic afflictions such as HIV and AIDS. It sheds light on what really is considered to be important to PLWHA, and not what the medical profile depicts. This assessment will also help clinicians to make judgements about areas of need of PLWHA and will be of assistance in planning interventions to address these needs with the overall aim of improving quality of life.

## Ethical considerations

Permission to conduct the study at the Antiretroviral Clinic, UCH, was granted by the University of Ibadan and/or University College Hospital Joint Institutional Review Board. The proposal was further reviewed by a committee set up by the management of the UCH ARV clinic to ensure compliance with the ethical and programme guidelines, which had been set up with the implementing partner funded by the President's Emergency Plan for AIDS Relief (PEPFAR). Both verbal and written informed consents were obtained from each participant before the questionnaire was administered.

## Method

### Study site and participant

The study site was the Antiretroviral (ARV) clinic at the University College Hospital in Ibadan, Nigeria. As of June 2008 when the study commenced, the clinic had recruited more than 9000 PLWHA, of whom 2500 were on highly active antiretroviral therapy (HAART). The study was conducted between June and September 2008.

### Study design

It was a cross-sectional study that involved 150 HIV-positive patients randomly selected with the Microsoft Excel 2003 random number generator from the register with the list of daily clinic appointments. The sample size estimation was determined from the prevalence of people expected to have acceptable quality of life scores amongst PLWHA, and not the prevalence of HIV infection in the environment.^22^

Furthermore, this report formed part of a larger study that compared the perception of QOL, in HIV-positive patients with HIV-negative patients, who were accessing care in the same centre. The patients were aged 18 years and older, were regular attendees of the clinic for at least 3 months, and had undergone some forms of psychosocial, nutritional and adherence counselling sessions; some were on HAART and some were not. All the patients who displayed significant evidence of depression or who were very ill were excluded from the study. All the patients were taken through the structured clinical interview for DSM-IV AXIS 1 Disorder (SCID) module by the Research Physician.

The pre-ART services offered at the ARV clinic, UCH Ibadan, include a comprehensive health care plan, which is initiated before the diagnosis of HIV sero-positive status, through to diagnosis, to the implementation of therapeutic activities. This comprehensive care incudes:

- psychological support. The provision of pre-counselling, post-counselling and ongoing counselling is part of the structure of the ART clinic, UCH. Every client is assigned to a counsellor who provides longitudinal care. In view of the collectivistic African culture, the benefit of group counselling sessions which reassures the clients that they have shared problems, forms a forum for reassurance and networking and hence improves their sense of social inclusion. A support group for PLWHA exists within the premises of the hospital and holds a meeting once a month, in addition to the provision of Home Based Care for its members with the support of the clinical staff at the same clinic. Clients may choose to enrol also in a number of adjunct support groups registered in the clinic and situated closer to the clients’ homes for easy accessibility, which encourages them to have treatment partners who help to monitor their adherence to ART when it is instituted. The support group also provides an avenue for social networking that is taken up more or less according to the dictates of the clients. This social network has assisted in psychological support and on rare occasions has led to more binding social unions such as matrimony. PLWHA are also helped through organised counselling sessions to disclose their sero-positive status to their spouses if, and when, it seems difficult to do this themself.
- medical care. The ARV clinic provides the full spectrum of care, from diagnosis of HIV and HIV-associated medical conditions and opportunistic infections, to the laboratory monitoring of HIV and AIDS with CD4, HIV RNA viral load, blood chemistries and haematology, completely free of charge. Management of HIV-related opportunistic infections, access to HIV-related drugs, prophylaxis for opportunistic infection, hospitalisation, nutritional support, interventions to reduce mother–to-child transmission (prevention of mother-to-child transmission of HIV and AIDS, or PMTCT), and health education amongst others, are also provided at no cost to the client.
- socio-economic support. The clinic does not provide financial support to clients even though this is very often requested. There is, however, a link to services funded explicitly for job creation for PLWHA. The clinic meets urgent financial requirements through the Indigent Fund, managed by the clinic staff. Waivers for hospital specialist consultations, specialised laboratory services and surgical procedures, are granted by the Management of UCH through the policy of the Federal Government of Nigeria. There are Quality Improvement activities in the clinic to ensure comprehensive health care involving PLWHA in service planning and delivery. The PLWHA actively participate in counselling, share positive experiences and encourage new clients, in addition to their management of support group activities and home-based care.

### Study instruments

An interviewer-administered questionnaire was used to collect data on sociodemographic profiles, quality of life, satisfaction with perceived social support, and clinical detail from medical records for the clinical HIV staging.

HIV staging was defined as:

- HIV asymptomatic (persons with no HIV-related symptoms)
- HIV symptomatic (persons with minor signs of HIV infection)
- AIDS (persons with major signs of disease such as weight loss, prolonged fever).

Respondents were grouped later into asymptomatic and symptomatic patients, based on this staging after recruitment for the study. Clinical examinations for HIV-related symptoms were carried out using a symptom checklist. Perceived social support was measured with the multidimensional scale of perceived social support (MSPSS).^23^ The MSPSS measures satisfaction with support from family members, significant others and friends. The QOL was assessed with the English version WHOQOL-Bref instrument;^7^ it was translated to Yoruba, the major local language in south-west Nigeria, and then it was back-translated into English. The Yoruba version was used for the small number of respondents who did not understand English. In this study, the WHOQOL-Bref consists of 26 items, with each item using a 5-point Likert scale. It has a Cronbach alpha of 0.80, which implies a high internal reliability. Higher scores depict better QOL. These items are distributed in four domains which are:

- physical domain. (This comprises 7 items that assess areas such as the presence of pain and discomfort, dependence on substances or treatments, energy and fatigue, mobility, sleep and rest, activities of daily living, and perceived working capacity.)
- psychological well-being (This comprises 8 items that assess areas such as patient's affect, both positive and negative, self-concept, higher cognitive functions; body image and spirituality.)
- social relationships (There are 3 items that assess areas such as social contacts, family support and the ability to care for family, and sexual activity.)
- environment (This comprises 8 items that assess aspects such as freedom, quality of home environment, physical safety and security and financial status, involvement in recreational activity, and health and social care as applicable to the quality and accessibility thereof.)

There are also 2 items that were examined separately: one which questioned the individual's overall perception of QOL, and the other which asked about the individual's overall perception of his or her health; however, these 2 items were not reported in this study. Domain scores are scaled in a positive direction (higher scores denote a higher quality of life). The mean score of items within each domain is used to calculate the domain scores compatible with the scores used in WHOQOL-100, and it is subsequently transformed to a 0–100 scale according to the WHO guideline.^7, 9, 18, 24^

### Statistical analysis

Data entry and analysis were performed with the statistical package for social science (SPSS)^25^ software, version 16.0. Categorical variables were summarised through the use of proportions, and continuous variables that included QOL scores with means and standard deviation. A further comparison was drawn through cross-tabulation of the variables. The student t-test was used for the analysis of statistical differences between the mean scores of QOL for dichotomous variables. Spearman's correlation was calculated to assess the effect of perceived social support on mean QOL scores of the respondents. The level of statistical significance was set at *p* < 0.05.

## Results

### Study participants

Of the 150 PLWHA who participated in the study, 93 (62.0%) were female and 57 (38.0%) were male, and the mean age was 38.1 ± 9 years (range 20–67 years). One hundred and twelve (74.7%) participants had no HIV-related symptoms at point of interview, while thirty-eight (25.3%) had symptoms that included dry or productive cough, itchy skin, body rashes, and a white patch in the mouth. One hundred and twelve (75.0%) participants had been aware of their HIV-positive status for more than 12 months, HIV diagnosis ranged 4–96 months (Table 1).

**TABLE 1:** Distribution of participants studied according to sex and presence of HIV-related symptoms, Ibadan, Nigeria 2008.

Variable	*N* = 150	

	*n*	%
**Sex**		
Male	57	38.0
Female	93	62.0
**HIV-related symptom status**		
Symptomatic	38	25.3
Asymptomatic	112	74.7
**Duration of HIV diagnosis**		
< 12 months	38	25.0
> 12 months	112	75.0

*Source*: Authors’ original data

*N*, Total number of participants; *n*, Given as a means of number.

The mean age of male participants was significantly higher than that of female participants, 41 ± 8.5 years versus 36.0 ± 9.0 years, *p* = 0.000. Female participants had a higher mean CD4 T-lymphocyte count, 407.0 ± 185.0 cells /mm^3^, than male participants, 329.0 ± 185.0 cells / mm^3^, *p* = 0.005 (Table 2).

**TABLE 2:** Mean age and mean CD4 T cell count of participants according to sex, Ibadan, Nigeria 2008.

Variable (Mean ± s.d.)	Male	Female	χ^2^	*p-*value
Age (years)	41 ± 8.5	36.0 ± 9.0	3.648	0.000
CD4 count (cells/mm^3^)	329.0 ± 185.0	407.0 ± 185.0	- 2.433	0.005†

*Source*: Authors’ original data

s.d., standard deviation; χ^2^, Chi-square.

† Mann-Whitney Test.

### Mean Quality of Life scores of participants

The mean scores of QOL (Table 3) were higher in the psychological domain (74.47 ± 13.94) followed by physical domain (71.60 ± 18.39) and environmental domains (70.10 ± 11.96), but the lowest score was observed in the social domain (63.83 ± 18.84).

**TABLE 3:** Distribution of means and standard deviations of transformed Quality of Life scores obtained from the quality of life questionnaire (World Health Organisation Quality of Life-Bref), Ibadan, Nigeria 2008.

QOL Domains	Mean scores (transformed 0–100) ± s.d.
Physical	71.6 0 ± 18.39
Psychological	74.47 ± 13.94
Social	63.83 ± 18.84
Environmental	70.10 ± 11.96

*Source*: Authors’ original data

QOL, Quality of Life; s.d., standard deviation.

Asymptomatic HIV-positive patients had significant better scores (Table 4) compared to symptomatic patients in the psychological domains (76.09 ± 12.93 versus 69.74 ± 15.79, *p* = 0.015) and physical domains (74.04 ± 16.85 versus 64.47 ± 20.94, *p* = 0.005).

**TABLE 4:** Quality of life domain scores of asymptomatic and symptomatic HIV-positive participants obtained from WHOQOL-Bref questionnaire, Ibadan, Nigeria 2008.

QOL Domains	(Mean QOL score ± s.d.)		*t*-value	*p*-value

	Asymptomatic	Symptomatic		
Physical	74.04 ± 16.85	64.47 ± 20.94	2.832	0.005
Psychological	76.09 ± 12.93	69.74 ± 15.79	2.466	0.015
Social	64.06 ± 18.62	63.16 ± 19.72	2.555	0.799
Environmental	70.54 ± 11.78	68.75 ± 12.52	0.795	0.428

*Source*: Authors’ original data

QOL, Quality of Life; s.d., standard deviation.

Male study participants had similar mean QOL scores as female participants in all four domains assessed (Table 5).

**TABLE 5:** Mean Quality of Life scores of male and female study participants, measured with world health organisation quality of life-Bref, Ibadan, Nigeria 2008.

QOL Domains	(Mean QOL score ± s.d.)		*t*-value	*p*-value

	Male	Female		
Physical	74.77 ± 17.43	74.87 ± 17.43	1.430	0.976
Psychological	74.36 ± 16.56	69.93 ± 19.30	0.617	0.155
Social	75.37 ± 13.32	73.91 ± 14.35	1.070	0.538
Environmental	65.94 ± 19.05	62.55 ± 18.70	1.264	0.286

*Source*: Authors’ original data

QOL, Quality of Life; s.d., standard deviation.

The perceived social support of the respondents is significantly correlated with all domains of QOL except with the physical domain (Table 6).

**TABLE 6:** Correlation analysis showing influence of perceived social support on mean quality of life scores of HIV-positive patients in Ibadan, 2008.

Quality of Life Domain	Spearman correlation coefficient ρ	*p*-value
Physical	0.13	0.115
Psychological	0.19	0.023
Social	0.35	0.000
Environmental	0.38	0.000

*Source*: Authors’ original data

## Discussion

The mean QOL scores are the highest in the psychological health domain, which measures the patient's affect, positive and negative self-concepts, higher cognitive functions, body image and spirituality. This is followed by the physical health domains which assess the presence of pain and discomfort, the dependence on medication, energy and fatigue, mobility, sleep and rest, activities of daily living, and perceived working capacity, which indicate a better quality of life in these domains. The environmental domain measures the patient's freedom, quality of home environment, financial status, quality and accessibility of health and social care. PLWHA has a higher score in this domain, which also indicates a better quality of life from what is seen in the social relation domains where the patient has the lowest mean scores, which indicates poorer QOL. The social relation domain measures aspects such as social contacts, family support and ability to look after family, and satisfaction with sexual activity.

These findings are similar to what Fatiregun reported in a similar study conducted in Kogi state, north-central Nigeria, in which the participants reported better mean QOL scores in the psychological and physical domains, but lower scores in the environmental and social domains.16 This finding is similar to what was documented in Brasilia where the majority of the PLWHA had been on HAART for a long time.^26^ In this study, the majority of the participants was diagnosed for over 1 year which has allowed them to adjust psychologically to the new way of life.

The lower score findings in the social domain may be a reflection of the stigmatisation and discrimination that HIV-positive patients are still exposed to in developing countries, with Nigeria no exception to the rule. This is contrary, however, to what an Indian study^15^ reported in which the PLWHA studied had the highest mean QOL scores in the social relations domain, which did not show any significant association with any of the determinants of QOL measured. Religious belief in reincarnation and the imperative call to behave well towards others to ensure reincarnation in a higher realm may account for the positive attitude amongst the Indian populace.

The correlation analysis on the degree of satisfaction with social support, on mean QOL domain scores of PLWHA studied, shows that significant association exists between perceived social support and quality of life scores in all domains except the physical domain. The correlation coefficients are the strongest between social support and the environmental domain, and between the social support and social relations domain of the respondents. This also confirms the report of Adedimeji's study.^19^

In this study asymptomatic patients who have higher mean QOL scores compared to their symptomatic counterparts in the physical and psychological domains, indicate better QOL in these domains which is similar to an earlier study where significant differences in QOL scores were observed in the physical health domain of patients who were asymptomatic, symptomatic, as well as those with AIDS.^15^ It reflects the impact of HIV and AIDS on the physical and psychological health of patients as the disease progresses. Another study reported a negative correlation of all WHOQOL-Bref domains with the number and intensity of patients’ symptoms,^26^ which this study was not designed to address.

The mean age of male participants was higher than that of their female counterparts, whereas the mean CD4 T cell count of female participants was higher than that of their male counterparts. This probably reflects the fact that female participants tend to have a better health-seeking behaviour and attitude to treatment compared to male participants, female patients are more likely to adhere to antiretroviral therapy, and they tend to be more informed on nourishing foods which will boost their immunity; they are also less likely to consume alcohol and smoke cigarettes. All of these reasons may contribute to the higher CD4 T cell count reported in the female group. Furthermore, although less likely, a higher CD4 T cell count may be expected in younger individuals, because thymic function is expected to be higher in the younger age group.^26^ It is interesting that there is no difference in the mean QOL scores of male PLWHA compared to female PLWHA in all domains assessed, which is similar to what Wig and his colleagues reported in a study in India.15 Most quality of life studies documented gender differences in QOL scores, that is, men have better QOL scores in some domains compared to women.^26–28^ In such studies, men actually reported higher QOL scores in the environmental domain and women had higher scores on the spirituality-or-religion and personal beliefs domain,^28^ suggestive of the fact that women tend to be more spiritually inclined than men, but the spirituality-or-religion domain was not assessed in the current study. The lack of sex differences in QOL scores reported in this study may also be a reflection of the demographic profiles of the study participants because there were more female participants than male participants. It could be an eye opener to the way African women perceive different aspects of their life which may be quite different from what women from other cultures portray, thus leaving room for further studies. This may also explain why the total support of the patient is best correlated with the environmental and social domains of quality of life, and the lesser correlation with their psychological domain scores, which is to be expected because this domain assesses aspects of self-concept, higher cognitive functions, body image and spirituality.

### Limitations and recommendation

The small sample size in the study was estimated on the assumption that not many PLWHA would have a good perception of their quality of life; future studies should involve a larger sample size. As a consequence, the result of this study cannot be generalised across Nigeria, because not all PLWHA are exposed to every intervention and supportive service available to PLWHA at the ARV clinic, UCH Ibadan; different ARV centres have different implementing partners and different mandates in terms of service delivery. A recommendation would be to have a multi-centred study across Nigeria that measures QOL in its different domains, while the services available in the different treatment programs are kept in mind, to establish with more certainty that the provision of a comprehensive range of support services is in fact the cause of the improved QOL in the different domains identified in the current study. The social environment across the country with its different cultural and religious characteristics would throw more light on the relationship between the social domain of QOL and client satisfaction, because factors external to the service delivery might be even more important in the impact on the social domain of QOL.

## Conclusion

The better QOL in the physical, psychological and environmental domains may be reflective of the effectiveness of some of the interventions PLWHA are exposed to at the ARV clinic, UCH Ibadan (on-going psychotherapy, free antiretroviral drugs). Poor social domain scores may suggest ineffectiveness of the social services network presently on the ground, because PLWHA are still exposed to stigmatisation and discrimination. If social support is improved, therefore, quality of life will improve further.
